# Preparation and Characterization of Multi-Doped Porous Carbon Nanofibers from Carbonization in Different Atmospheres and Their Oxygen Electrocatalytic Properties Research

**DOI:** 10.3390/nano12050832

**Published:** 2022-03-01

**Authors:** Tao Wang, Oluwafunmilola Ola, Malcom Frimpong Dapaah, Yuhao Lu, Qijian Niu, Liang Cheng, Nannan Wang, Yanqiu Zhu

**Affiliations:** 1 Key Laboratory of Modern Agriculture Equipment and Technology, School of Agricultural Engineering, Jiangsu University, Zhenjiang 212013, China; wangt073@163.com (T.W.); luyuhao1999@163.com (Y.L.); 2 Advanced Materials Research Group, University of Nottingham, Nottingham NG7 2RD, UK; Oluwafunmilola.ola1@nottingham.ac.uk; 3 Institute of Environmental Health and Ecological Security, School of the Environment and SafetyEngineering, Jiangsu University, Zhenjiang 212013, China; 5103200301@stmail.ujs.edu.cn (M.F.D.); Clcheng@ujs.edu.cn (L.C.); 4 Guangxi Institute for Fullerene Technology, Key Laboratory of New Processing Technology for Nonferrous Metals and Materials, School of Resources Environment and Materials, University of Guangxi, Nanning 530000, China; wangnannan@gxu.edu.cn

**Keywords:** electrospinning, porous carbon nanofibers, oxygen reduction reaction, oxygen evolution reaction

## Abstract

Recently, electrocatalysts for oxygen reduction reaction (ORR) as well as oxygen evolution reaction (OER) hinged on electrospun nanofiber composites have attracted wide research attention. Transition metal elements and heteroatomic doping are important methods used to enhance their catalytic performances. Lately, the construction of electrocatalysts based on metal-organic framework (MOF) electrospun nanofibers has become a research hotspot. In this work, nickel-cobalt zeolitic imidazolate frameworks with different molar ratios (Ni_x_Co_y_-ZIFs) were synthesized in an aqueous solution, followed by Ni_x_Co_y_-ZIFs/polyacrylonitrile (PAN) electrospun nanofiber precursors, which were prepared by a simple electrospinning method. Bimetal (Ni-Co) porous carbon nanofiber catalysts doped with nitrogen, oxygen, and sulfur elements were obtained at high-temperature carbonization treatment in different atmospheres (argon (Ar), Air, and hydrogen sulfide (H_2_S)), respectively. The morphological properties, structures, and composition were characterized by scanning electron microscopy (SEM), transmission electron microscopy (TEM), selected area electron diffraction (SAED), X-ray diffraction (XRD), and X-ray photoelectron spectroscopy (XPS). Moreover, the specific surface area of materials and their pore size distribution was characterized by Brunauer-Emmett-Teller (BET). Linear sweep voltammetry curves investigated catalyst performances towards oxygen reduction and evolution reactions. Importantly, Ni_1_Co_2_-ZIFs/PAN-Ar yielded the best ORR activity, whereas Ni_1_Co_1_-ZIFs/PAN-Air exhibited the best OER performance. This work provides significant guidance for the preparation and characterization of multi-doped porous carbon nanofibers carbonized in different atmospheres.

## 1. Introduction

Water splitting, metal-air batteries, and fuel cells are notable renewable energy technologies that rely heavily on oxygen reduction reaction (ORR) and oxygen evolution reaction (OER). Nonetheless, the slow kinetics of oxygen evolution and reduction reaction impedes their energy conversion efficiency [1]. Therefore, developing various oxygen reduction together with oxygen evolution catalysts is very important to improve their efficiencies. At present, Pt/C and RuO_2_/IrO_2_ are the main commercial catalysts serving as ORR and OER catalysts [2,3]. However, they are both precious metal catalysts with a high price, low resource reserves, and low stability. In recent years, researchers have developed a variety of novel catalysts, mainly to reduce the use of precious metals to build non-precious metal catalysts. Among them, transition metal-carbon matrix composite catalysts have been widely studied. Many carbon-based materials (carbon nanoparticles [4], biochar [5], graphene [6], carbon nanotubes [7], carbon nanofiber [8], etc.) have become an important carrier for the construction of oxygen electrocatalysts because of their wide sources, being cheap and easy to obtain, their good electrical conductivity and diverse structures [9]. Transition metal (Fe, Co, Ni, Cu, Zn, etc.) or heteroatomic (N, P, S, O, etc.) doping is a common approach for preparing these catalysts [10,11]. Moreover, the performance and functionalization of the catalysts were improved through single doping to multiple doping. Moreover, conversion between different forms of compounds from simple compounds to oxides [12], hydroxides [13], carbides [14], sulfides [15], phosphide [16], and their hybrids have achieved good catalytic performances.

Electrospinning is a novel technology for preparing one-dimensional nanofibers [17]. Electrospun nanofibers have been commonly utilized in many domains due to their merits, such as high void fraction, large specific surface area, large aspect ratio, and small diameter [18]. Recently, the construction of oxygen electrocatalysts based on electrospun nanofibers has attracted much attention [19]. Many electrospun nanofiber catalysts with different properties were prepared by transition metal and heteroatomic doping. The commonly used methods include direct pyrolysis of inorganic salts in electrospun nanofibers and in-situ surface growth of electrospun nanofibers. However, these methods tend to cause agglomeration of doped nanoparticles, or the preparation steps are relatively complicated. Metal-organic frameworks (MOFs) are crystalline materials fabricated by merging metal ions and organic ligands through coordination bonds [20,21,22,23]. A variety of high-performance oxygen electrocatalysts were prepared by changing the composition and carbonization conditions of MOF materials [24].

To date, some MOF-based composites have shown promising OER and ORR properties [25,26,27]. Further combination with electrospun nanofibers is beneficial for highly distributed active sites and porous carbon nanofiber catalysts preparation. The OER and ORR properties of bimetallic MOF electrospun structures show good dual-function performance [28]. However, carbonization of the same precursor in different atmospheres and conversion of different phases have not been reported. It is paramount to study the structure, composition, and properties of catalytic materials under different carbonization atmospheres for high-performance catalyst development.

Herein we report, for the first time, the assessment of bi-metal (nickel-cobalt) zeolitic imidazolate frameworks (NiCo-ZIFs) electrospun carbon nanofibers under three different atmospheres in one work. The different molar ratios of the bimetallic nanocrystal materials (Ni_x_Co_y_-ZIFs) were synthesized in an aqueous solution, and then Ni_x_Co_y_-ZIFs/polyacrylonitrile (PAN) nanofiber precursors were prepared by simple electrospinning method. Afterwards, the multi-doped porous carbon nanofiber catalysts for OER and ORR were obtained by carbonization in different atmospheres (Ar, Air, and H_2_S). The morphologies, structures, crystal compositions, and elemental compositions of the precursors and derived catalysts after carbonization were characterized. Finally, the OER and ORR catalytic effects of various samples under different conditions were investigated.

## 2. Experimental Section

### 2.1. Materials

Ethanol (≥99.7%), N,N-dimethylformamide (DMF, ≥99.5%), Co(NO_3_)_2_·6H_2_O (99%), potassium hydroxide (KOH, 98%), Ni(NO_3_)_2_·6H_2_O (98%), polyacrylonitrile (PAN, molecular weight of 150 Kg.mol^−1^), and 2-methylimidazole (C_4_H_6_N_2_, MIM) were obtained from Aladdin Biochemical Technology Co., Ltd. (Shanghai, China). DuPont Co. supplied the Nafion solution (5 wt%). The purchased chemicals were utilized as received without additional purification unless specified in this work.

### 2.2. Preparation of Bimetal Ni_x_Co_y_-ZIFs Nanocrystals

Ni_x_Co_y_-ZIFs nanocrystals were prepared by adding various molar ratios (1, 2, and 4 mmol) of Ni(NO_3_)_2_·6H_2_O and Co(NO_3_)_2_·6H_2_O (4 mmol) to 2-methylimidazole (300 mmol) aqueous solution (100 mL) before continuous stirring at room temperature (RT) for 24 h [29]. This was accompanied by 10 min centrifugation and rinsing thrice with deionized (DI) water. Ni_x_Co_y_-ZIFs powders were obtained, with x and y representing n(Ni)/n(Co) molar ratio.

### 2.3. Preparation of Bimetal NixCoy-ZIFs/PAN Nanofibers

1.0 g of as-prepared Ni_x_Co_y_-ZIFs nanoparticles, 0.5 g of PAN polymer, and 4.5 g of DMF solvent were mixed to prepare the electrospinning solution after stirring for several hours. The obtained mixture was transferred to a plastic syringe (5.0 mL) with a single nozzle (stainless steel) of 0.6 mm diameter. For the typical electrospinning procedure, the applied high voltage with set interval between the collector (aluminum foil) and tip were 20 kV and 15 cm, respectively. Moreover, the syringe injection speed was 0.6 mL·h^−1^. After electrospinning, nanofibers from the aluminum foil were placed in a vacuum oven set to 80 °C overnight for residual solvent removal [30].

### 2.4. Preparation of Multi-Doped Porous Carbon Nanofibers

The dried Ni_x_Co_y_-ZIFs/PAN nanofibers were heated in different atmospheres in a tube furnace. Three main cases were considered; (i) in Ar atmosphere: the Ni_x_Co_y_-ZIFs/PAN precursor was heated at 800 °C for 2 h with 5 °C.min^−1^ heating rate, before cooling to room temperature; (ii) in Air atmosphere: after a carbonization process consistent with Ar atmosphere, the oxidation process was completed by heating for 2 h at 300 °C in an Air atmosphere with 5 °C.min^−1^ heating rate; (iii) in H_2_S atmosphere: during a carbonization process consistent with Ar atmosphere, the sulfurization process was initiated when the temperature rose to 800 °C, and H_2_S gas was supplied at this temperature for 2 h continuously.

### 2.5. Material Characterization

The morphological features of the synthesized catalysts were characterized by scanning electron microscopy (SEM) (JSM-6701F, JEOL, Tokyo, Japan) and transmission electron microscopy (TEM) (Tecnai G2 20 S-T win, FEI, Hillsboro, United States). The crystal structures were characterized with a D8 Advance X-ray diffraction (XRD) unit with Cu Kα radiation (Bruker, Billerica, United States ). Elemental composition and doping state were characterized by an ESCLAB 250 X-ray photoelectron spectroscopy (XPS) (Thermo Fischer Scientific, Massachusetts, USA). Pore size distribution and corresponding Brunauer-Emmett-Teller (BET) surface area were characterized using an Autosorb-iQ gas sorptometer (Quantachrome, Florida, USA) by the standard volumetric procedure [31].

### 2.6. Electrochemical Measurements

For electrochemical performance measurements in the prepared samples, all were done in a conventional three-electrode system (CHI 660C and 760E, Chenhua, Shanghai, China), in which a Pt wire, an Ag/AgCl electrode (3.0 M KCl solution), and a GCE (glassy carbon electrode, d = 4.0 mm) functioned as the counter electrode, reference electrode, and the working electrode, respectively. For ORR and OER, 0.1 mol·L^−1^ and 1.0 mol·L^−1^ KOH aqueous electrolytes at 25 °C were used. On catalyst ink synthesis, the catalyst (5.0 mg) was distributed in a water/ethanol (*v*/*v* = 4:1) solution (1.0 mL) with added Nafion solution (5.0 wt%, 5.0 μL) using ultrasonication for 0.5 h. Afterward, catalyst ink (5.0 μL) was spread onto the working electrode surface for further electrochemical measurements. The catalytic activities of OER and ORR were characterized by linear sweep voltammetry (LSV) curves set at 10 mV·s^−1^ scan rates [32]. A homemade Zn–air battery was fabricated to examine the potential–current polarization curves [30]. The experiments were done in triplicates.

## 3. Results and Discussions

**Figure 1:**
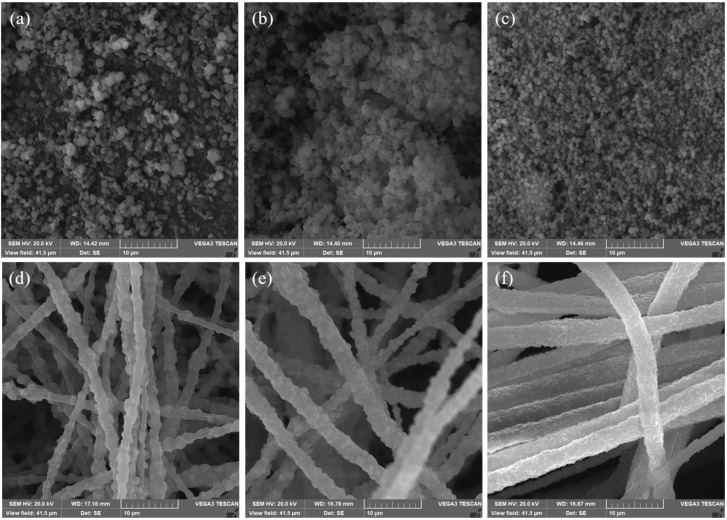
The SEM images of the prepared nickel-cobalt zeolitic imidazolate frameworks (Ni_x_Co_y_-ZIFs) crystals and Ni_x_Co_y_-ZIFs/polyacrylonitrile (PAN) nanofibers at various molar ratios: (**a**) Ni_1_Co_1_-ZIFs; (**b**) Ni_1_Co_2_-ZIFs; (**c**) Ni_1_Co_4_-ZIFs; (**d**) Ni_1_Co_1_-ZIFs /PAN; (**e**) Ni_1_Co_2_-ZIFs/PAN; (**f**) Ni_1_Co_4_-ZIFs/PAN nanofibers.

The micro-morphologies of synthesized bimetallic Ni_x_Co_y_-ZIFs and the Ni_x_Co_y_-ZIFs/PAN nanofiber precursors were characterized by SEM images, as depicted in Figure 1. From Figure 1a–c, the bimetallic Ni_x_Co_y_-ZIFs appeared in the form of nanoparticles. With the increase of Co ratio, the morphology of nanoparticles became smaller as the diameter gradually decreased from Figure 1a–c. As shown in Figure 1d–f, the Ni_x_Co_y_-ZIFs/PAN nanofibers had one-dimensional structures, with Ni_x_Co_y_-ZIFs convex-like crystals coating its surface. In Figure 1f, the diameters of Ni_1_Co_4_-ZIFs/PAN nanofibers were more uniform due to the small diameter of the coated Ni_1_Co_4_-ZIFs particles.

**Figure 2:**
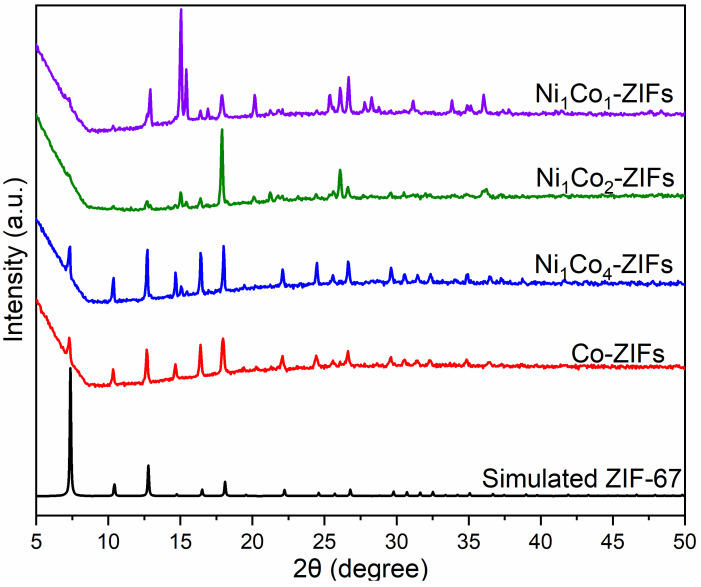
The XRD patterns of different Ni_x_Co_y_-ZIFs crystals: Ni_1_Co_1_-ZIFs; Ni_1_Co_2_-ZIFs; Ni_1_Co_4_-ZIFs, and Co-ZIFs.

The crystal structure of the synthesized bimetallic Ni_x_Co_y_-ZIFs nanoparticles was characterized by XRD patterns illustrated in Figure 2. With the increase of Ni content, the crystal structure gradually disappeared. However, the crystal structure of Ni_1_Co_4_-ZIFs was the same as the Co-ZIFs. This was in consent with already reported works [29].

**Figure 3:**
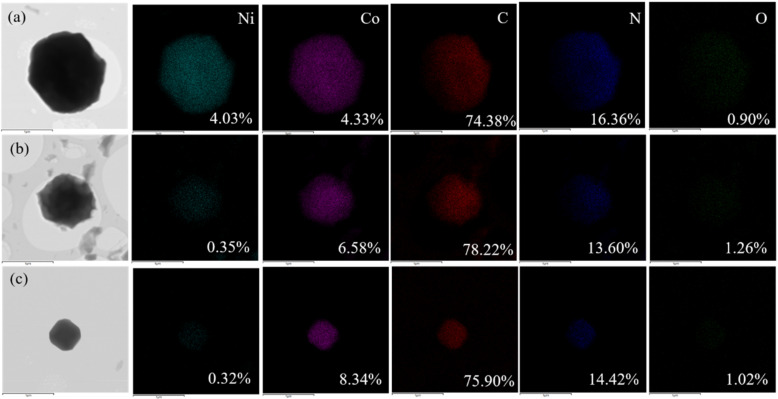
The TEM images and different elemental mappings of Ni_x_Co_y_-ZIFs crystals: (**a**) Ni_1_Co_1_-ZIFs; (**b**) Ni_1_Co_2_-ZIFs; (**c**) Ni_1_Co_4_-ZIFs.

The content and distribution of various elements in the synthesized bimetallic Ni_x_Co_y_-ZIFs nanoparticles were studied by TEM elemental mappings in Figure 3. The contents of C, N, and O elements in bimetallic Ni_x_Co_y_-ZIFs crystals had no significant difference. However, the proportion of Ni and Co was quite different. In Ni_1_Co_1_-ZIFs crystals, the Ni:Co ratio was close to the theoretical value. However, in Ni_1_Co_2_-ZIFs and Ni_1_Co_4_-ZIFs samples, the doping ratio of Ni was far lower than the theoretical value, though the content was similar. Combined with XRD patterns, it was proven that Ni doping would affect the crystal stability, and Ni ions could hardly enter the crystal skeleton when the content of Co ions was high.

**Figure 4:**
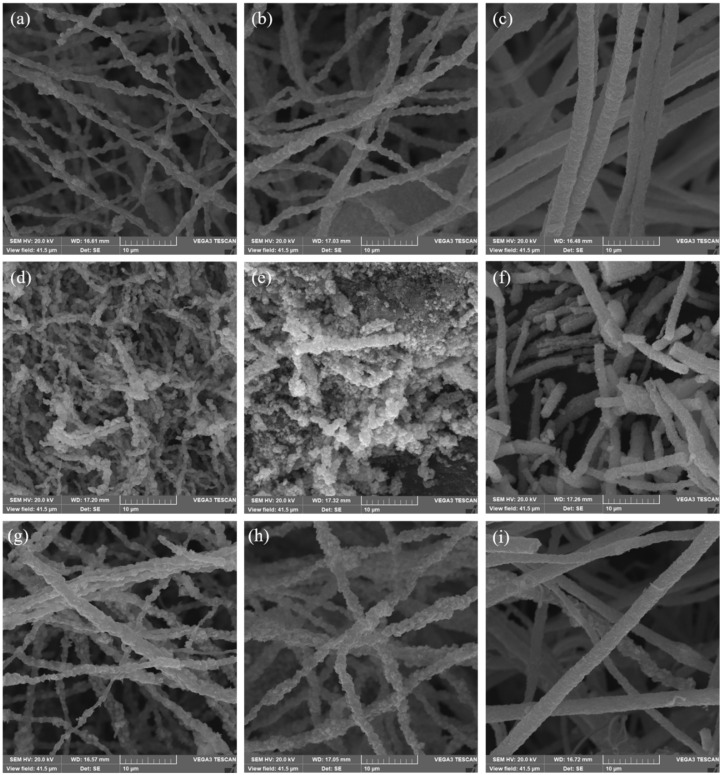
The SEM images of multi-doped porous carbon nanofibers derived from different Ni_x_Co_y_-ZIFs/PAN nanofibers by carbonization, oxidation, and sulfurization in different atmospheres (Ar, Air, and H_2_S): (**a**) Ni_1_Co_1_-ZIFs/PAN-Ar, (**b**) Ni_1_Co_2_-ZIFs/PAN-Ar, (**c**) Ni_1_Co_4_-ZIFs/PAN-Ar; (**d**) Ni_1_Co_1_-ZIFs/PAN-Air, (**e**) Ni_1_Co_2_-ZIFs/PAN-Air, (**f**) Ni_1_Co_4_-ZIFs/PAN-Air; (**g**) Ni_1_Co_1_-ZIFs/PAN-H_2_S, (**h**) Ni_1_Co_2_-ZIFs/PAN- H_2_S, (**i**) Ni_1_Co_4_-ZIFs/PAN- H_2_S.

The microstructures of the varied samples under different atmosphere carbonization were characterized by SEM images shown in Figure 4. The nanofiber samples that were carbonized in Ar atmosphere maintained the rough surface morphology. After carbonization in Air, some nanofibers were found to be broken. During the carbonization process, the morphology of the nanofibers was the same as those under Ar gas after the sulfurization process. The results showed that the oxidation process after carbonization easily led to the destruction of the overall structure of the nanofiber. In contrast, the general morphology of the nanofiber did not change significantly during the carbonization process under the protection of inert Ar gas.

**Figure 5:**
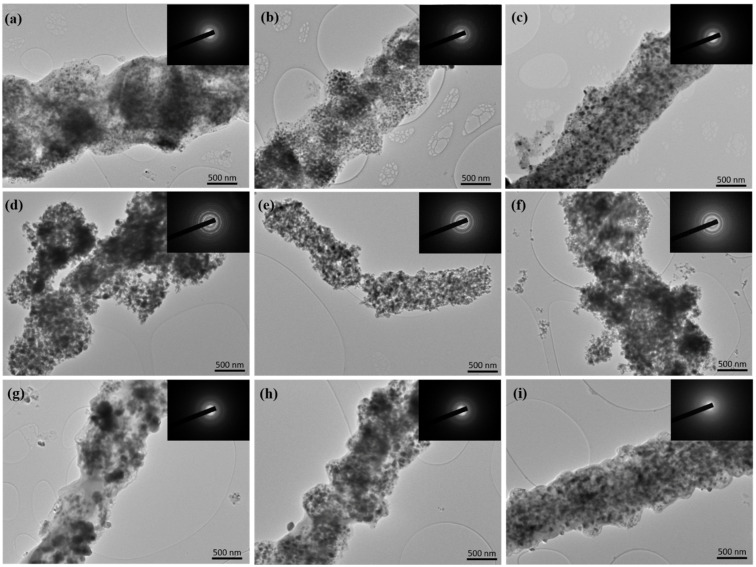
The TEM images of multi-doped porous carbon nanofibers derived from different Ni_x_Co_y_-ZIFs/PAN nanofibers by carbonization, oxidation and sulfurization in different atmospheres (Ar, Air, and H_2_S): (**a**) Ni_1_Co_1_-ZIFs/PAN-Ar, (**b**) Ni_1_Co_2_-ZIFs/PAN-Ar, (**c**) Ni_1_Co_4_-ZIFs/PAN-Ar; (**d**) Ni_1_Co_1_-ZIFs/PAN-Air, (**e**) Ni_1_Co_2_-ZIFs/PAN-Air, (**f**) Ni_1_Co_4_-ZIFs/PAN-Air; (**g**) Ni_1_Co_1_-ZIFs/PAN- H_2_S, (**h**) Ni_1_Co_2_-ZIFs/PAN- H_2_S, (**i**) Ni_1_Co_4_-ZIFs/PAN- H_2_S.

The internal structure of carbonized samples was additionally characterized by TEM images, as displayed in Figure 5. Many metal nanoparticles were observed on nanofibers after carbonization in Ar atmosphere (Figure 5a–c). Before carbonization, the outline of the Ni_x_Co_y_-ZIFs crystals was much prominent. The nanoparticles derived from Ni_1_Co_4_-ZIFs/PAN precursors were relatively small and uniform. After oxidation in Air, the derived nanoparticles became larger, and some nanofibers broke (Figure 5d–f). The obtained results were coherent with the SEM images. After the sulfurization process, agglomeration of nanoparticles occurred. However, the morphology and distinct pore structure were still maintained (Figure 5g–i). From the crystal diffraction pattern (Figure 5 inserted), it was observed that the samples obtained by carbonization in different atmospheres had good crystal structure, among which the crystal diffraction ring structure after oxidation was the most distinct. At the same time, the catalysts derived from Ni_1_Co_4_-ZIFs/PAN had uniform nanoparticle doping and a complete nanofiber structure.

**Figure 6:**
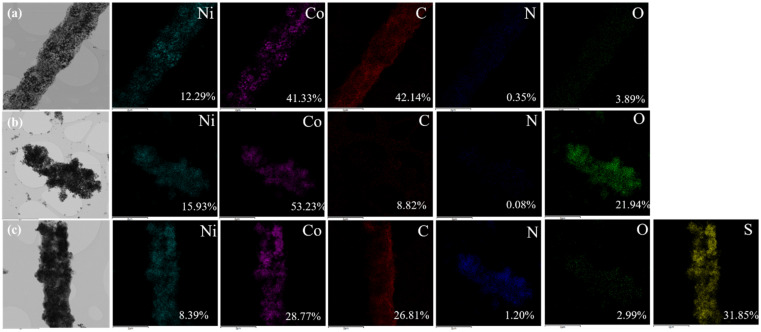
The TEM images and elemental mappings of multi-doped porous carbon nanofibers derived from Ni_1_Co_4_-ZIFs/PAN nanofibers by (**a**) carbonization, (**b**) oxidation, and (**c**) sulfurization in different atmospheres (Ar, Air, and H_2_S) at 800 °C.

Furthermore, the TEM images and elemental mappings of multi-doped porous carbon nanofibers derived from Ni_1_Co_4_-ZIFs/PAN nanofibers by carbonization, oxidation, and sulfurization in different atmospheres (Ar, Air, and H_2_S) at 800 °C is shown in Figure 6. After carbonization, all the ratio of Ni and Co was close to the theoretical ratio before carbonization. During carbonization in only Ar atmosphere, the carbon element content remained at 42.14%. After oxidation treatment in the Air, oxygen content increased as expected to 21.94%, while carbon content decreased to 8.82%, which may be due to the generation of carbon dioxide. After the sulfurization process, it was found that there were a lot of sulfur elements up to about 31.85%.

**Figure 7:**
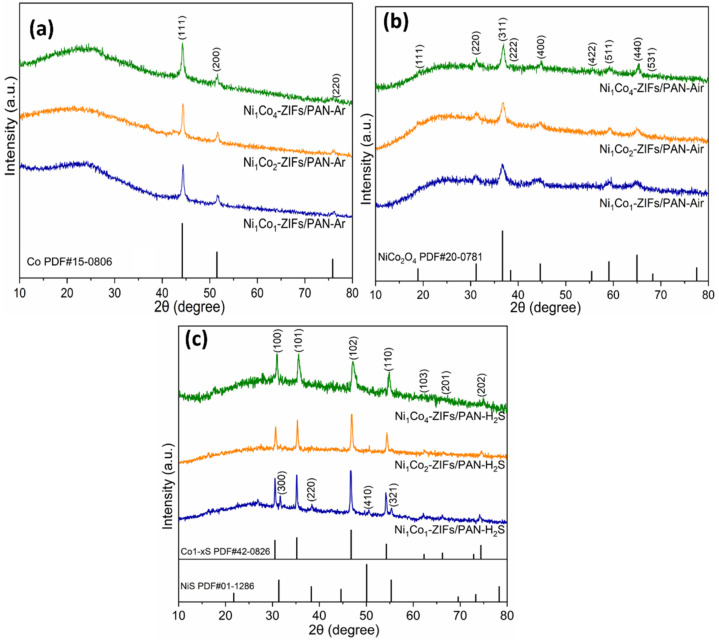
The XRD patterns of multi-doped porous carbon nanofibers derived from different Ni_x_Co_y_-ZIFs/PAN nanofibers by (**a**) carbonization, (**b**) oxidation, and (**c**) sulfurization in different atmospheres (Ar, Air, and H_2_S) at 800 °C.

From Figure 7, the XRD characterization was done to study the crystal composition changes in different carbonization atmospheres. For Ni_x_Co_y_-ZIFs/PAN-Ar, the XRD pattern showed the diffraction peaks of two main metallic elements at 44.5° and 51.8°, which proved that Ni_x_Co_y_ alloy metal doping was realized [33]. Examining Ni_x_Co_y_-ZIFs/PAN-Air, the XRD pattern revealed diffraction peaks of multiple metal oxides at 36.8°, 44.3°,59.8°, and 65.0°, indicating Ni_x_Co_y_ metal oxides doping was achieved [34,35]. Moreover, the XRD patterns of Ni_x_Co_y_-ZIFs/PAN-H_2_S offered Ni_x_Co_y_ metallic sulfide diffraction peaks at 30.8°, 35.1°, 47.2°, and 54.7°, which affirmed multiple metallic sulfide doping. Hence, the results above demonstrated that carbonization could be used to prepare catalyst materials with various doping types in different atmospheres [29,36]. The weak and broad peak observed around 26.2° indicated that very little graphitic carbon was present [37,38].

**Figure 8:**
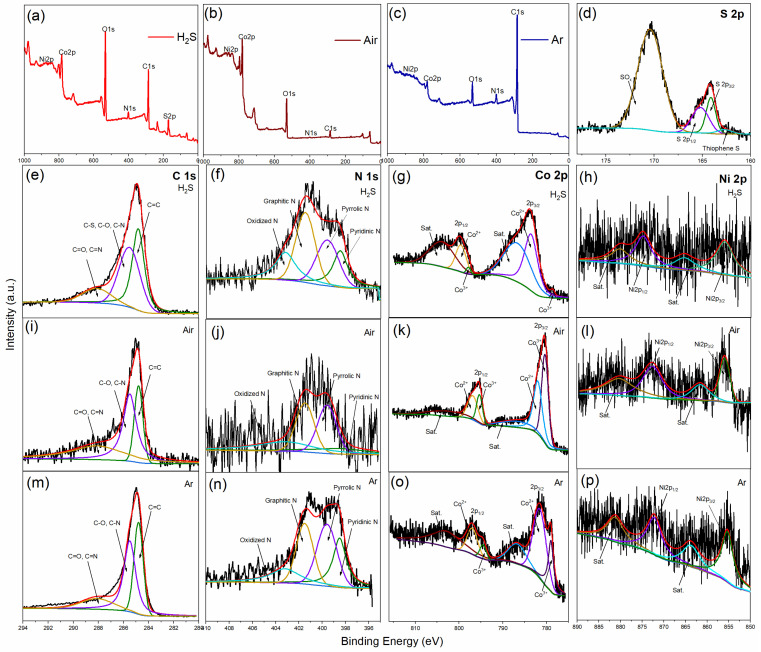
X-ray photoelectron spectrum of Ni_1_Co_4_-ZIFs/PAN nanofiber under different atmospheres (Ar, Air, and H_2_S); Survey spectrum (**a**–**c**), S 2p (**d**), C 1s (**e**,**i**,**m**), N 1s (**f**,**j**,**n**), Co 2p (**g**,**k**,**o**), and Ni 2p (**h**,**l**,**p**).

The Ni_1_Co_4_-ZIFs/PAN nanofiber was then selected for further characterizations under different atmospheres because of its higher crystallinity and structural stability according to XRD, SEM, and TEM-EDS earlier discussed. As depicted in Figure 8a–c, the XPS spectrum of the Ni_1_Co_4_-ZIFs/PAN material contains Ni, Co, C, N, O, and S (under H_2_S only) elements. The S 2p spectrum (Figure 8d), from the H_2_S atmosphere, offered peaks at 170.4, 162.6, 165.2, and 164.1 eV, which corresponded to SOx, thiophene S, 2p_1/2_, and 2p_3/2_ in CoS, respectively [31,39]. For C 1s (Figure 8e,i,m), the peaks at 284.8, 285.5, and 288.1 eV were identified as C=C, saturated carbon (C−S, C−O, C−N), and unsaturated carbon species (C=O, C=N) respectively [31,40]. The N1s spectrum (Figure 8f,j,n) was represented by four components at 403.2, 401.5, 399.6, and 398.5 eV, denoting oxidized N, graphitic N, pyrrolic N, and pyridinic N [31,41].

Moreover, the Co 2p spectrum had 2p_1/2_ and 2p_3/2_ components, as shown in Figure 8g. Under H_2_S exposure, the obtained peaks at 799.5 and 783.7 eV were related to the Co^2+^ species [42], whereas those at 778.7 and 797.8 eV were for Co^3+^, respectively [43,44]. In Air (Figure 8k), the peaks at 795.3 and 780.4 eV were attributed to Co^3+^, while those at 782 and 796.8 eV were for Co^2+^, respectively [45]. The satellite components appeared at 802.9 and 786.7 eV [46]. Under Ar (Figure 8o), the peaks at 796.9 and 781.6 eV were characterized as Co^2+^, while those at 794.5 and 779 eV were assigned to Co^3+^ species [43,45].

Considering Ni 2p under H_2_S atmosphere (Figure 8h), the Ni2p_1/2_ and Ni2p_3/2_ peaks were observed at 874.7 and 856 eV, respectively [47]. From Figure 8l, Ni2p_1/2_ and Ni2p_3/2_ peaks were identified at 872.6 and 856 eV, besides the two shake-up satellites. Under Ar (Figure 8p), these main peaks occurred at 872.3 and 855.4 eV, respectively [48,49]. The increased binding energies witnessed in Co 2p and Ni 2p for the H_2_S atmosphere can be ascribed to higher oxidation characteristics occurring after S-doping [50].

**Figure 9:**
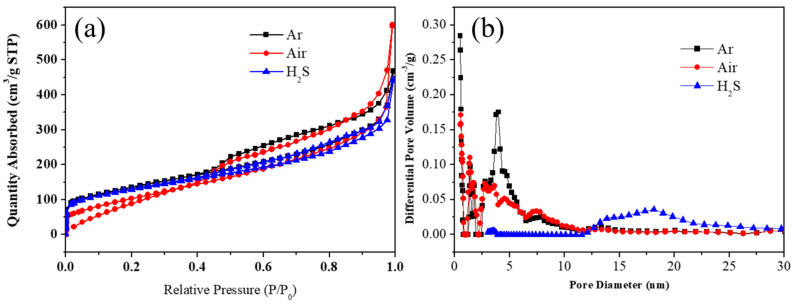
The (**a**) N_2_ sorption isotherm plots and (**b**) pore size distribution curves of multi-doped porous carbon nanofibers derived from different Ni_1_Co_4_-ZIFs/PAN nanofibers by carbonization, oxidation, and sulfurization in different atmospheres (Ar, Air, and H_2_S) at 800 °C.

The effects of different carbonization processes on specific surface area and porosity were examined using nitrogen sorption curves of the materials (Figure 9). The catalyst specific surface areas gained by carbonization in Ar, Air, and H_2_S atmosphere were 484.27, 489.37, and 461.70 m^2^ g^−1^, respectively. The pore size distribution was primarily centered at about 5 nm. After Air oxidation, it was found that the hysteresis region of nitrogen adsorption and desorption curve had no apparent change. The distribution was primarily concentrated below 10 nm. It was noted that the hysteresis of nitrogen adsorption and desorption curve decreased after sulfurization, whereas the distribution of the pore sizes increased to 15–20 nm. The pore structure and specific surface area tend to affect the catalytic performance of catalysts.

**Figure 10:**
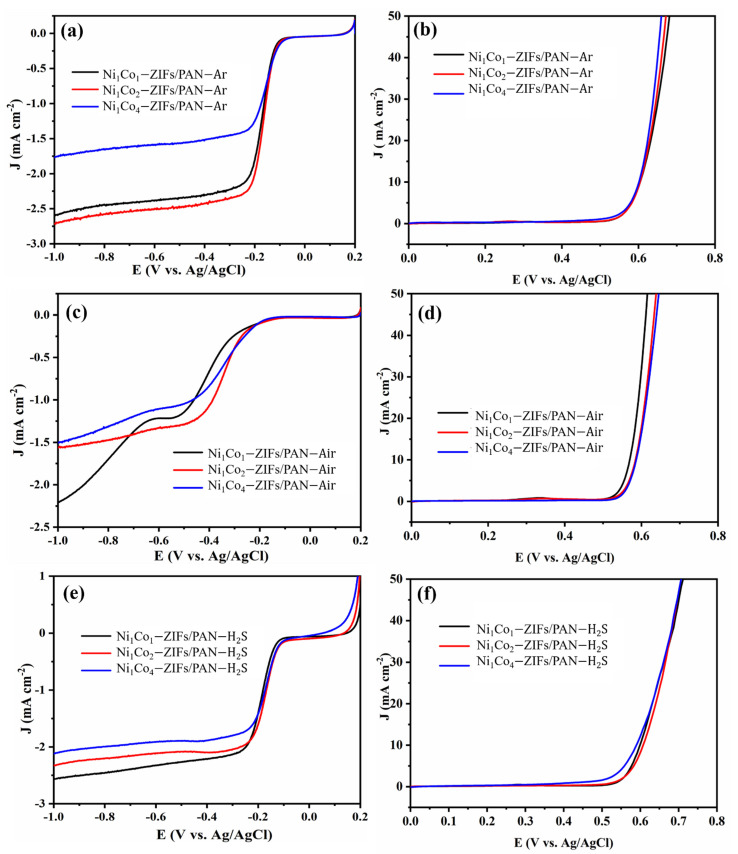
The ORR and OER performances of multi-doped porous carbon nanofibers derived from different Ni_x_Co_y_-ZIFs/PAN nanofibers by (**a**,**b**) carbonization, (**c**,**d**) oxidation, and (**e**,**f**) sulfurization in different atmospheres (Ar, Air, and H_2_S) at 800 °C.

The ORR and OER performances under different carbonization states were studied. As depicted in Figure 10a, the Ni_1_Co_2_-ZIFs/PAN-Ar sample had the best ORR output. The overall improvement in the Ar-carbonized sample towards ORR could be attributed to its higher pyridinic N amount compared to those in Air and H_2_S. This could boost ORR performance via a 4-electron pathway [51,52]. Moreover, the larger mesoporous arrangement in the Ar-carbonized samples increased the active sites for mass transfer, thereby inducing better electrochemical properties [53,54]. In terms of OER behavior in Ni_x_Co_y_-ZIFs/PAN-Ar samples (Figure 10b), there were no significant changes in potential value at 10 mA·cm^−2^. Figure 10c showed that the ORR of Ni_x_Co_y_-ZIFs/PAN-Air decreased after the oxidation process. However, their OER performances were significantly enhanced, especially for Ni_1_Co_1_-ZIFs/PAN-Air (Figure 10d). After the sulfurization process (Figure 9e), the ORR yields were better than oxidation but worse than carbonation products in the Ar atmosphere. After sulfurization, the OER performance of Ni_1_Co_4_-ZIFs/PAN-H_2_S was better than the other samples (Figure 10f). In addition, it was observed from Figure 10a,c,e that when the ratio of Ni/Co was 1/2, their ORR catalytic performance was better than the other ratios. Meanwhile, as illustrated in Figure 10b,f, when the ratio of Ni/Co was 1/4, their OER catalytic performances were better compared to the other ratios.

Moreover, the catalyst stability for Ni_1_Co_2_-ZIFs/PAN-Ar and Ni_1_Co_1_-ZIFs/PAN-Air were examined. The chronoamperometric measurement for 18,000 s (Figures S1 and S2) offered current retention values of 62.96% (ORR) and 33.80% (OER) for Ni_1_Co_2_-ZIFs/PAN-Ar and Ni_1_Co_1_-ZIFs/PAN-Air, respectively. Moreover, after using the best ORR catalyst, Ni_1_Co_2_-ZIFs/PAN, as oxygen cathodes in rechargeable Zn-Air batteries under the three atmospheres, Ar gave a higher peak power density of 41.96 mW·cm^−2^ (Figure S3).

The electrochemical impedance spectroscopy (EIS) was conducted for all the samples (Figure S4). This was carried out in 5 mM [Fe(CN)_6_]^3−/4−^ with 0.1 M KCl solutions at a bias potential of 0.23 V. The amplitude was set at 10 mV and the frequency ranged from 0.1 to 100 Hz. Similar outputs were observed in Ni_1_Co_2_-ZIFs/PAN and Ni_1_Co_4_-ZIFs/PAN materials under Air and H_2_S atmospheres. The most shifted Nyquist plot semi-circles witnessed for the atmospheres followed the order; Ni_1_Co_1_-ZIFs/PAN-Air > Ni_1_Co_4_-ZIFs/PAN-Ar > Ni_1_Co_1_-ZIFs/PAN-H_2_S. Moreover, the Koutecky–Levich (K-L) plot, deduced from polarization curves at different rotation rates (Figures S5–S7), Ni_x_Co_y_-ZIFs/PAN-Ar samples showed good linearity and this approximately implied first-order-reaction kinetics [55].

## 4. Conclusions

In summary, bimetallic Ni_x_Co_y_-ZIFs were prepared by hydrothermal synthesis. The best crystalline structure was achieved when the Ni/Co ratio was 1/4. One-dimensional Ni_x_Co_y_-ZIFs/PAN nanofiber precursors were synthesized by simple electrospinning. Subsequently, Ni_x_Co_y_ bimetallic compound doped porous carbon nanofiber catalysts with N, O, S doping were obtained by carbonization under different gas (Ar, Air, H_2_S) atmospheres, respectively. The morphology, structure, crystal composition, elemental content, and specific surface area of the carbonized catalysts were characterized by SEM, TEM, XRD, elemental mapping, and BET. The carbon content of the nanofiber decreased, and the nanoparticles agglomerated during the oxidation process, leading to nanofiber fracture. The S-doping reduced the specific surface area and increased the nanofiber pore size. Importantly, it was found that Ni_1_Co_2_-ZIFs/PAN-Ar and Ni_1_Co_1_-ZIFs/PAN-Air yielded the best results for ORR (−0.09 V vs. Ag/AgCl) and OER (0.57 V vs. Ag/AgCl) performances after the carbonization process, respectively. In addition, Ni_1_Co_4_-ZIFs/PAN-Ar offered better peak power density of 41.96 mW·cm^−2^.

## Data Availability

The data presented in this study are available on request from the corresponding author.

## Supplementary Materials

The following supporting information can be downloaded at: https://www.mdpi.com/article/10.3390/nano12050832/s1, Figure S1: Electrocatalytic stability of Ni_1_Co_2_-ZIFs/PAN-Ar in O_2_-saturated 0.1 M KOH aqueous solution at ™0.30 V (vs. Ag/AgCl); Figure S2: Electrocatalytic stability of Ni_1_Co_1_-ZIFs/PAN-Air in O_2_-saturated 1.0 M KOH aqueous solution at 0.60 V (vs. Ag/AgCl); Figure S3: Discharge polarization and power density curves of Zn–air batteries using Ni_1_Co_2_-ZIFs/PAN under (a) Ar (b) Air and (c) H_2_S as ORR catalysts (mass loading of 1.2 mg cm^-2^); Figure S4: EIS patterns of Ni_x_Co_y_-ZIFs/PAN nanofibers under different atmospheres (Ar, Air, and H_2_S) at 0.23 V; Figure S5: LSV curves of Ni_x_Co_y_-ZIFs/PAN nanofibers at different rotation speeds from 400 to 2025 rpm (a,c,e), K–L plots of Ni_x_Co_y_-ZIFs/PAN nanofibers under Ar atmosphere at different potentials (b,d,f); Figure S6: LSV curves of Ni_x_Co_y_-ZIFs/PAN nanofibers at different rotation speeds from 400 to 2025 rpm (a,c,e), K–L plots of Ni_x_Co_y_-ZIFs/PAN nanofibers under Air atmosphere at different potentials (b,d,f); Figure S7: LSV curves of Ni_x_Co_y_-ZIFs/PAN nanofibers at different rotation speeds from 400 to 2025 rpm (a,c,e), K–L plots of Ni_x_Co_y_-ZIFs/PAN nanofibers under H_2_S atmosphere at different potentials (b,d,f).
