# Supplementary material for: Preparation and Characterization of Multi-Doped Porous Carbon Nanofibers from Carbonization in Different Atmospheres and Their Oxygen Electrocatalytic Properties Research
Source: Nanomaterials (Basel). 2022 Mar 1;12(5):832. doi: 10.3390/nano12050832 (PMC8912686; doi:10.3390/nano12050832)
Supplement: Supplementary file 1 [file nanomaterials-12-00832-s001.zip › nanomaterials-1569051 supplementary.pdf]

Supporting information

Preparation and Characterization of Multi-doped Porous Carbon Nanofibers from Carbonization in Different Atmospheres and Their Oxygen Electrocatalytic Properties Research

Tao Wang [1], Oluwafunmilola Ola [2], Malcom Frimpong Dapaah [3], Yuhao Lu [1], Qijian Niu [1,*], Liang Cheng [3], Nannan Wang [4] and Yanqiu Zhu[4,*]

[1]Key Laboratory of Modern Agriculture Equipment and Technology, School of Agricultural Engineering, Jiangsu University, Zhenjiang, Jiangsu, 212013, China
[2]Advanced materials research group, University of Nottingham, Nottingham, NG7 2RD
[3]Institute of Environmental Health and Ecological Security, School of the Environment and Safety Engineering, Jiangsu University, Zhenjiang, 212013, China
[4]Guangxi institute for Fullerene Technology, Key Laboratory of New Processing Technology for Nonferrous Metals and Materials, School of Resources Environment and Materials, University of Guangxi, Nanning, Guangxi, 530000, China

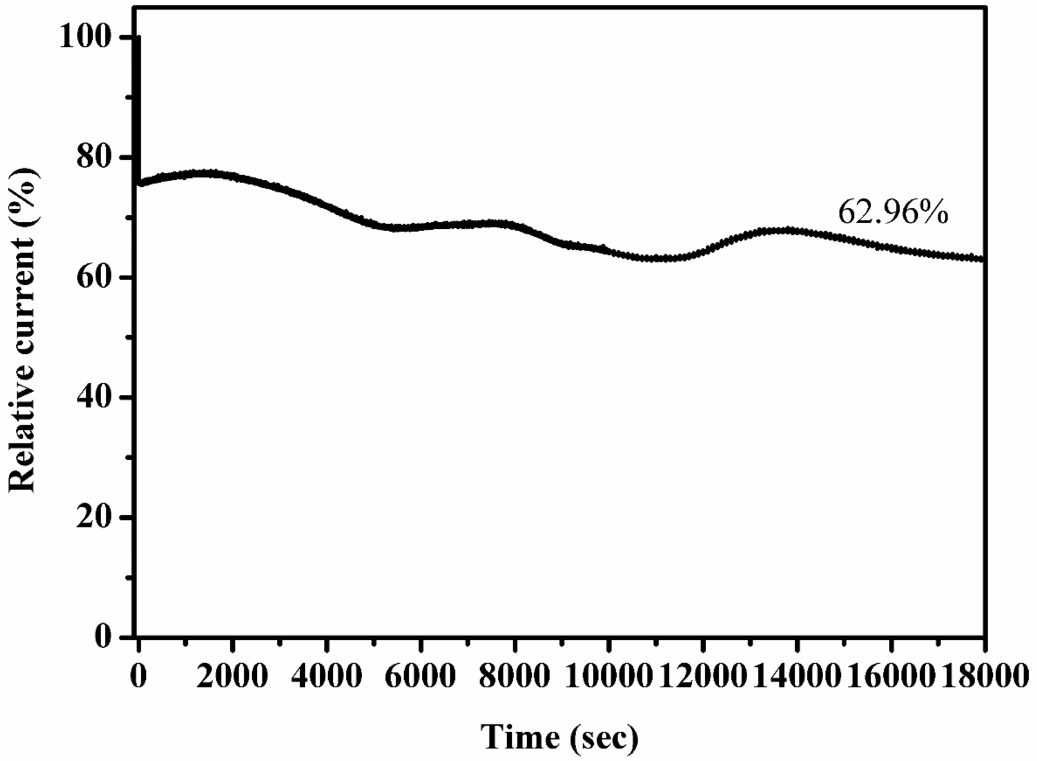

Figure S1 Electrocatalytic stability of Ni_1Co_2-ZIFs/PAN-Ar in O_2-saturated 0.1 M KOH aqueous solution at -0.30 V (vs. Ag/AgCl).

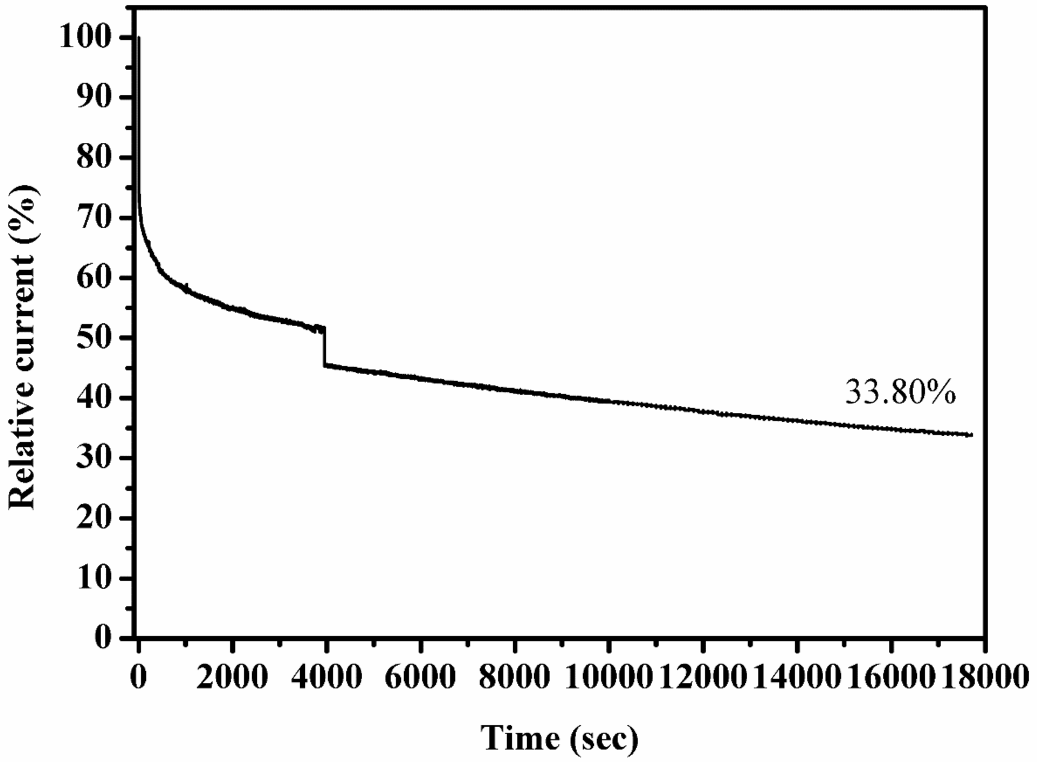

Figure S2. Electrocatalytic stability of Ni_1Co_1-ZIFs/PAN-Air in O_2-saturated 1.0 M KOH aqueous solution at 0.60 V (vs. Ag/AgCl).

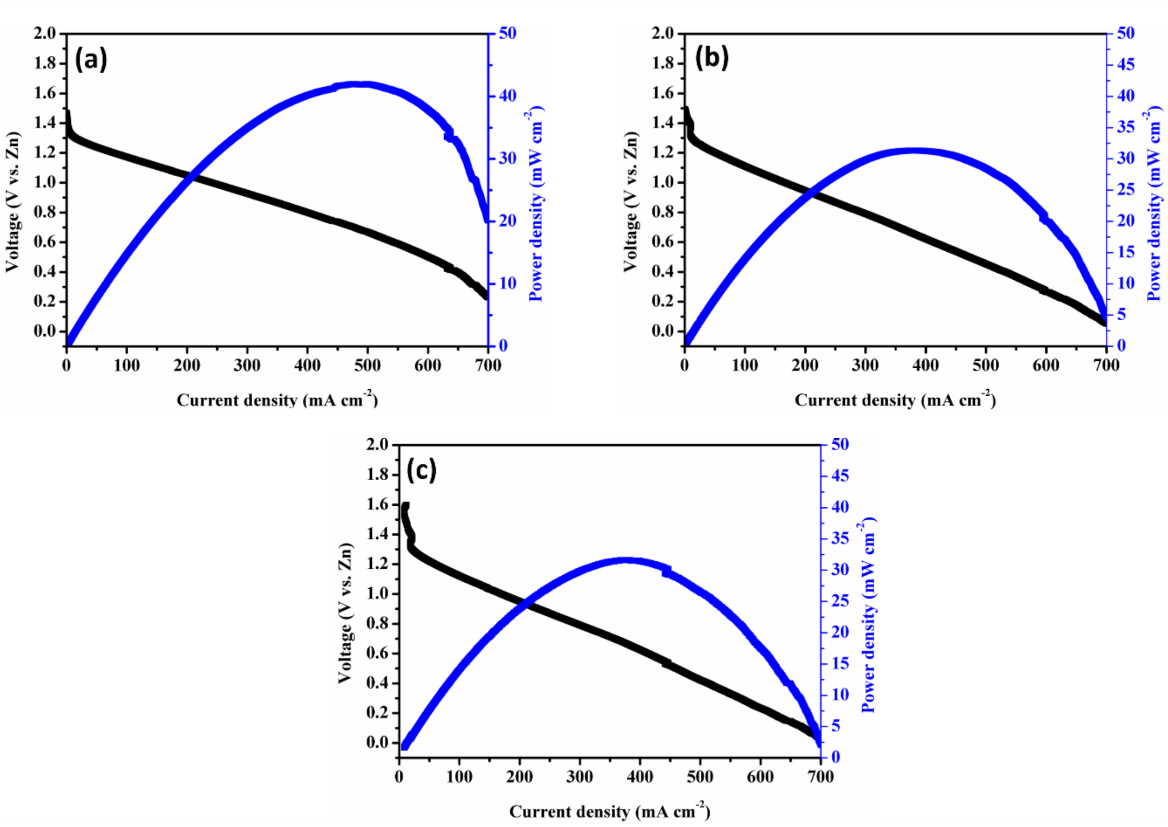

Figure S3 Discharge polarization and power density curves of Zn–air batteries using Ni_1Co_2-ZIFs/PAN under (a) Ar (b) Air and (c) H_2S as ORR catalysts (mass loading of 1.2 mg cm^{-2}).

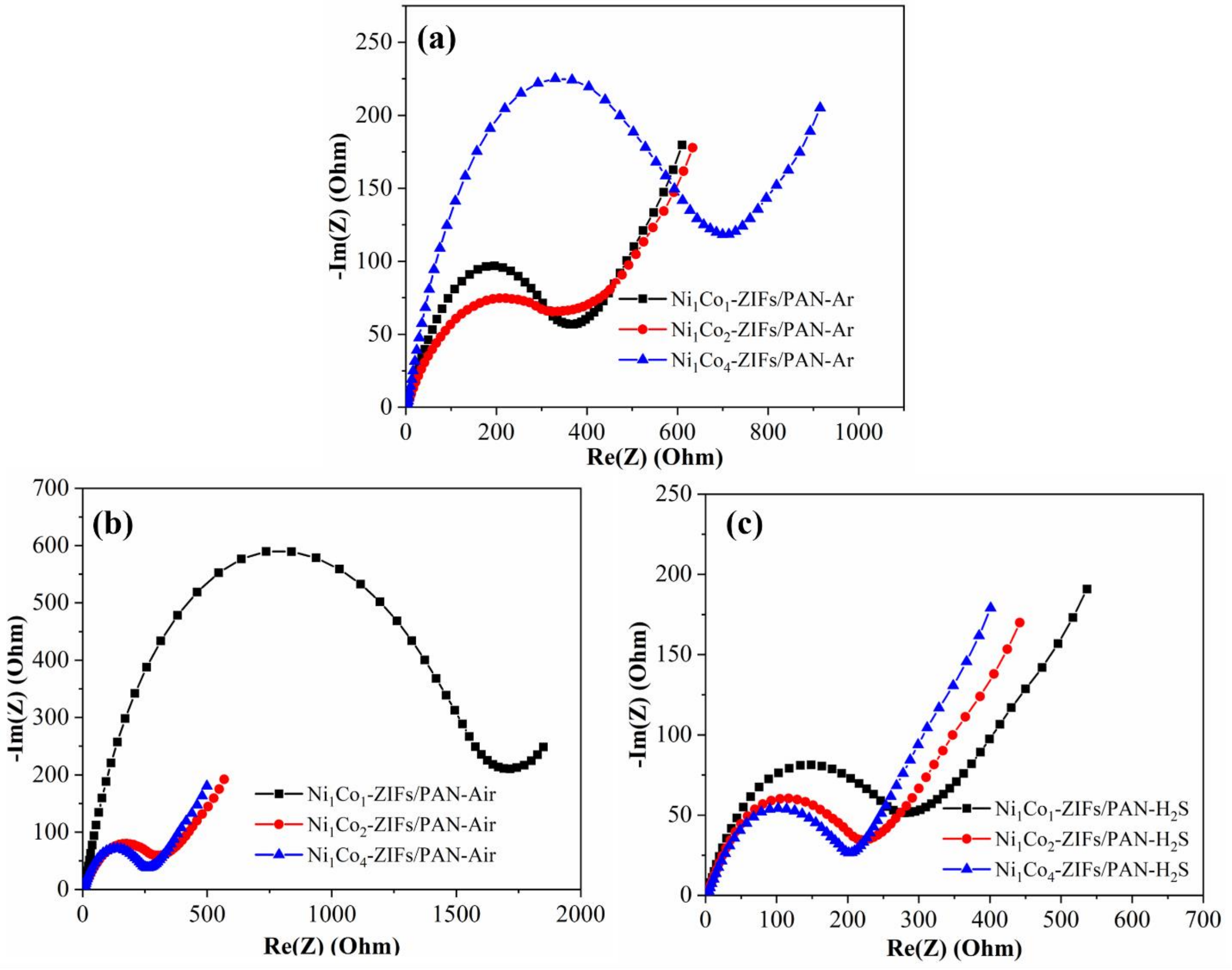

Fig. S4 EIS patterns of Ni_xCo_y-ZIFs/PAN nanofibers under different atmospheres (Ar, Air, and H_2S) at 0.23 V

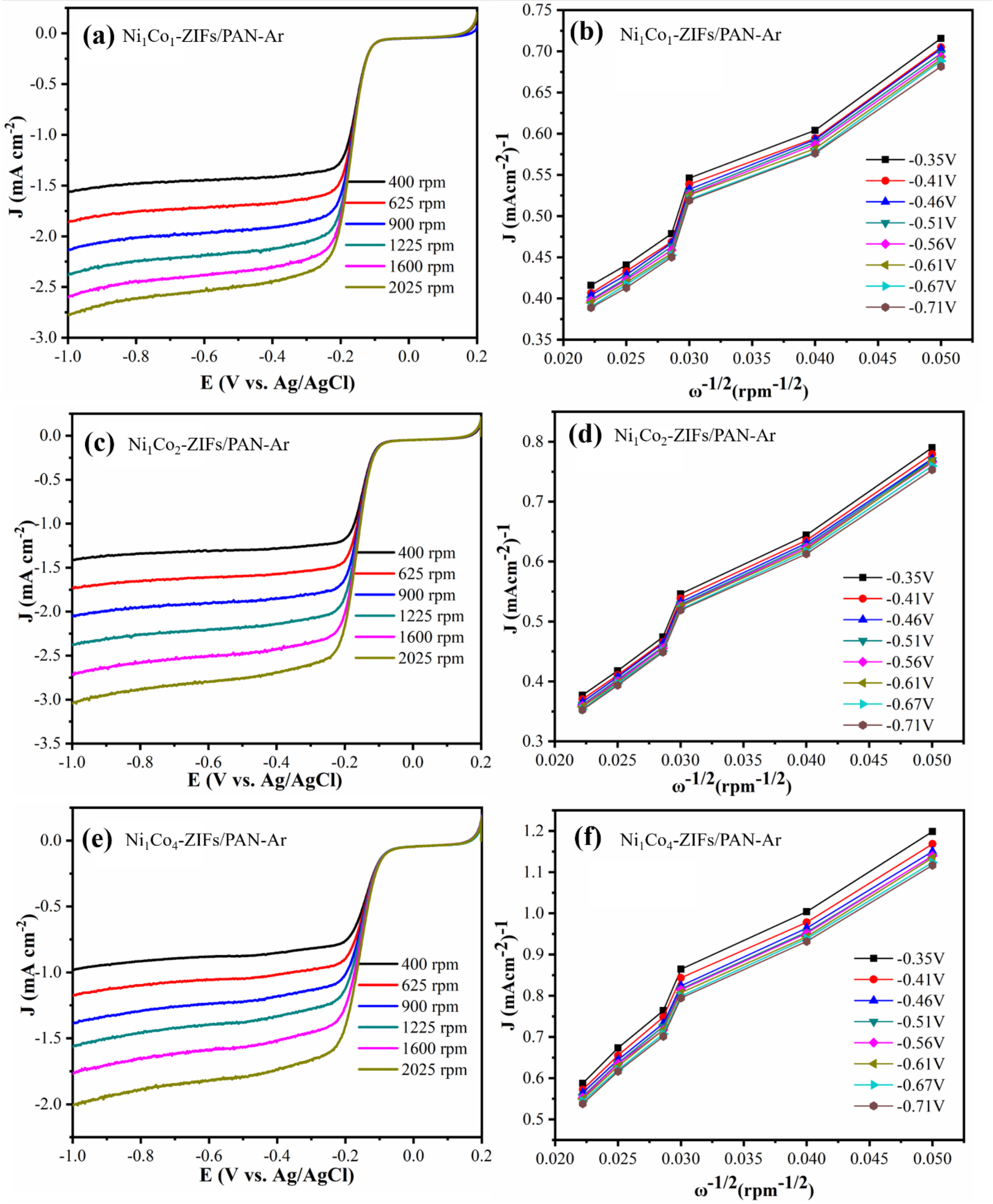

Fig. S5 LSV curves of Ni_xCo_y-ZIFs/PAN nanofibers at different rotation speeds from 400 to 2025 rpm (a,c,e), K–L plots of Ni_xCo_y-ZIFs/PAN nanofibers under Ar atmosphere at different potentials (b,d,f).

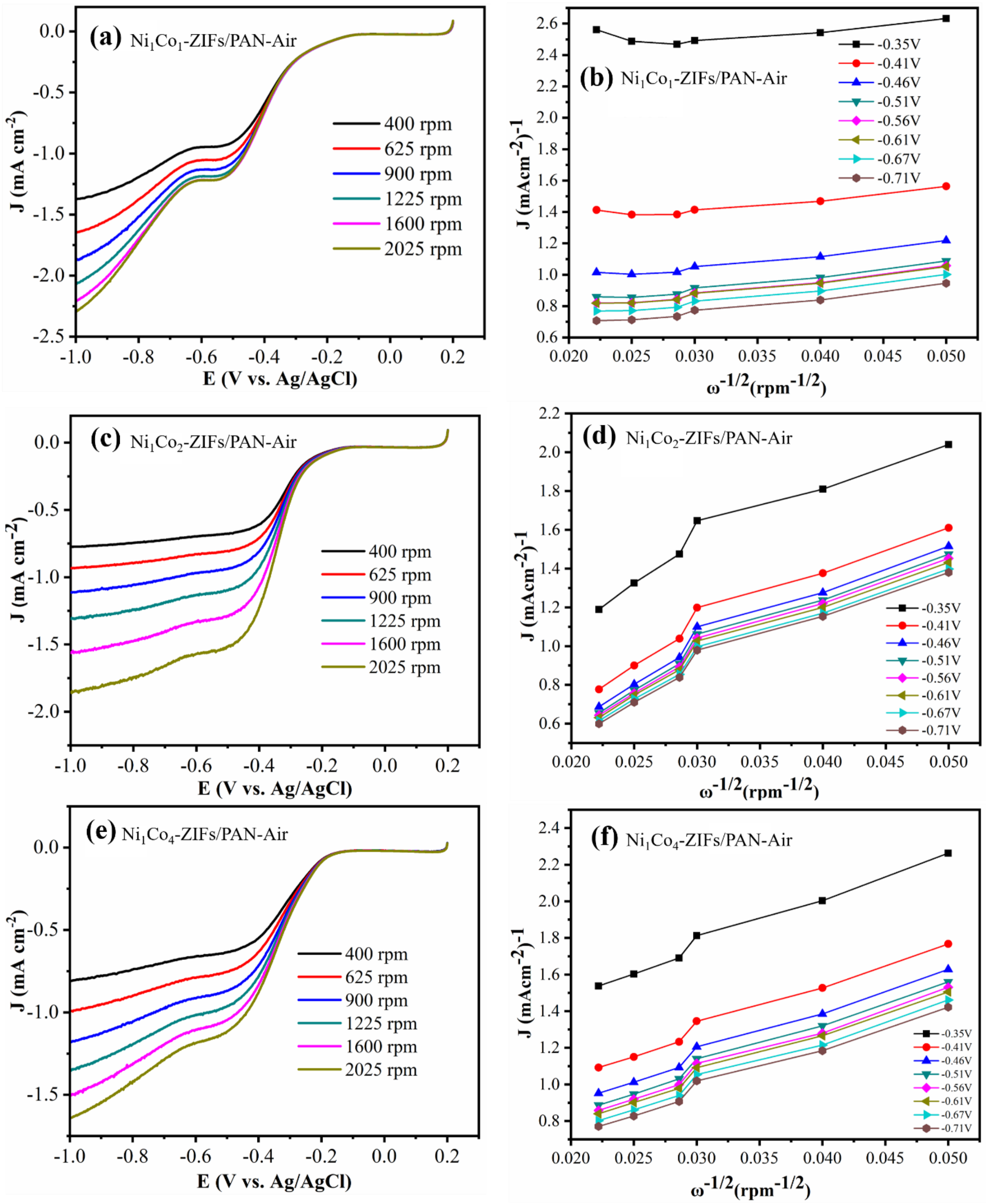

Fig. S6 LSV curves of Ni_xCo_y-ZIFs/PAN nanofibers at different rotation speeds from 400 to 2025 rpm (a,c,e), K–L plots of Ni_xCo_y-ZIFs/PAN nanofibers under Air atmosphere at different potentials (b,d,f).

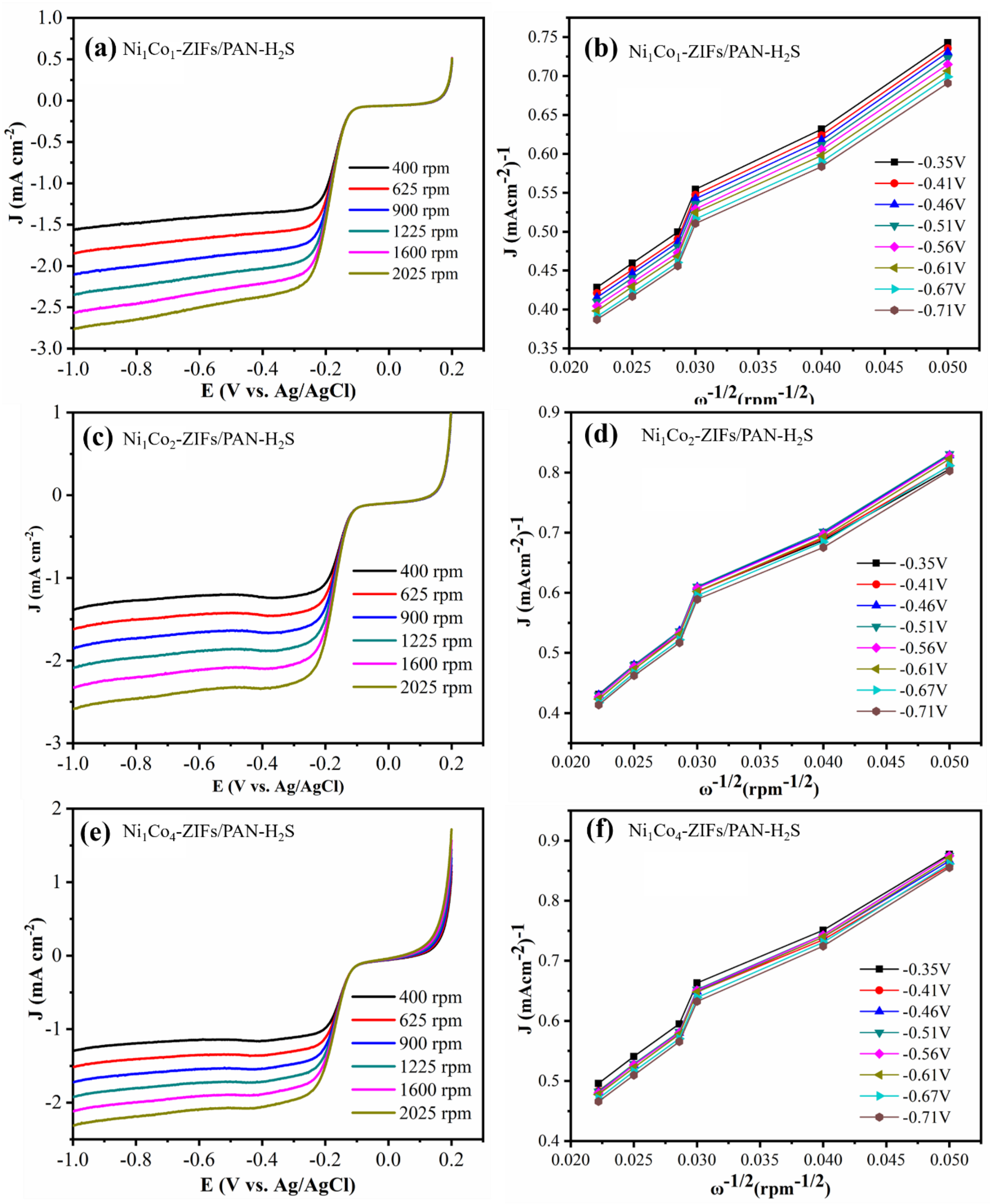

Fig. S7 LSV curves of Ni_xCo_y-ZIFs/PAN nanofibers at different rotation speeds from 400 to 2025 rpm (a,c,e), K–L plots of Ni_xCo_y-ZIFs/PAN nanofibers under H_2S atmosphere at different potentials (b,d,f).
